# Annotations

**Published:** 1898-01-15

**Authors:** 


### ANNOTATIONS.

### A "Finish Fight."

MANY comments have been made in the general press on an article which lately appeared in THE HOSPITAL, entitled "A Knock-out Blow." Of these, we will only refer to one—a letter in the *Belfast Evening Telegraph.* The writer of this letter maintains that in such cases as the one we mentioned it is the concussion of the head against the boards that causes the unconsciousness. We doubt this always being so. We do not, however, refer to the matter for the sake of arguing that question, but merely to draw the attention of our more quiet and stay-at-home readers to the description given by the writer of the sort of thing that happens in boxing contests, where the game is "to the finish," and the knock-out is allowed. The writer says: "The point-of-the-jaw-hit is usually put in from a semi-crouching position, whereby the striker raises himself upright at the termination of the blow, thus lifting his adversary clean off his feet, and causing him to fall almost upside down on to his head. This hit, if the person struck falls on to, say a gymnasium mat, will not put him to sleep, but a jerking motion of the head will ensue." In other words, the man will go into a fit. The distinction drawn is interesting. We believe, however, that in the case we referred to the man did fall on a gymnasium mat, and yet he was "put to sleep." The writer very properly protests against such "one-hit" matches, and says that boxing contests often consist of getting a couple of men, whose knowledge of the game is limited to a swing hit at "the point," or at "the mark," and putting them to gaze at each other until one of them is laid low in the manner indicated. A "finish fight," he says, is not sport—boxing is; all of which agrees with what we said as to the tendency of this power of finishing the match at any moment by a "knock-out" to encourage undue violence.

### Tyranny in Prison.

MR. TALLACK, secretary of the Howard Association, has done well to draw attention to the fact that, however humane may be the conduct of our prisons and the treatment of our prisoners so far as the higher officials are concerned, the actual treatment of the prisoners themselves is in the hands of men of a very different type. "It is not," he says, "the governor or deputy-governor of a prison who comes chiefly into contact with the prisoners, but the warders." That these men have it in their power to deal infinitely harshly with those in their charge is undoubted, and we cannot but agree that when cases of tyranny come to light they should be investigated in the fullest and most public manner possible. Mr. Tallack describes the case of a man named Cox, who, on being recently sent from a prison in Manchester to the Prestwich Lunatic Asylum, was found by the surgeons who examined him on his arrival to have had eight of his ribs broken; and he points out not only that considerable pressure had to be used to obtain an investigation of the case, but that when this took place it was conducted in secret, even the visiting justices not being allowed to be present at the first inquiry. Mr. Tallack also reminds us of the well-known case of the man Gatcliffe, who a few years ago died from violence sustained in the same gaol. This crime was never brought home to anyone, and it remains, as Mr. Tallack says, "presumptively, at least," a mystery to this day. But is it really a mystery, or was the matter hushed up? That is the question in regard to which Mr. Tallack's suggestions raise a not unreasonable anxiety. He says, "there is nearly always noticeable, amongst the superior officials, a natural tendency to hush up matters more or less. Even the Home Office is not altogether exempt from this tendency." We need not insist on the gravity of this charge. Our faith in our prison system entirely hinges on our faith in the superior officials exercising a most minute and suspicious supervision over the warders, and mercilessly exposing those who presume by their cruelty to increase the sentences of the judges. Yet we now find a man who somehow has got eight ribs broken while in gaol, we find a secret inquiry, and we are met with a statement from a well-informed source, that the very officials whom we have to trust have a tendency to hush matters up. The only cure we can see for this condition of affairs is the most absolute publicity in the investigation of all complaints.

### Tuberculosis in the Insane.

A PROPOSAL which comes dangerously near to much debateable ground in medical ethics is made by Dr. Eric France in a paper on "Tuberculosis in the Insane," published in the *Journal of Mental Science.* All modern conceptions of the etiology of tuberculosis tend to the conclusion that the prevalence of tuberculosis in asylums is due to the increased chances of infection to which people are exposed when large numbers are aggregated under one roof, under artificial conditions, under a certain amount of restraint as to exercise, and often under very unnatural conditions in regard to ventilation. The chance of an infection of this sort being insidiously spread among the inmates of asylums is greatly aggravated by the fact that lunatics in many cases take but small heed of their physical condition, and that they may be in an infective state for some time before the presence of phthisis is discovered. Moreover, among lunatics it is very difficult, if not impossible, to enforce those measures in regard to the disposal of the expectoration which are our most important means for preventing the spread of tuberculosis in institutions. What Dr. France proposes, then, is perfectly logical. He would isolate all who show signs of being affected, weeding the cases out as they occur. With this we think everyone will agree. But Dr. France, being a man of a logical turn of mind, sees the difficulty which stands in his way. It is clearly useless to isolate unless one can isolate all who are affected. The entire value of isolation consists, then, in "early and absolutely accurate diagnosis," and for the sake of obtaining such early information of the presence of the disease he would inoculate with tuberculin all those who come on the "suspected" list. Again, this is an absolutely logical sequence of the present position of our knowledge on the subject; and when the injection of tuberculin comes into general use for purposes of diagnosis outside asylums, as it already has done in veterinary practice, then we shall not have a word to say about it. Till then we would ask whether ethical questions do not arise in regard to the use of this means of diagnosis on the insane in our asylums?

